# Supplementary material for: Effect of Peptide Receptor Radionuclide Therapy in Combination with Temozolomide against Tumor Angiogenesis in a Glioblastoma Model
Source: Cancers (Basel). 2021 Oct 8;13(19):5029. doi: 10.3390/cancers13195029 (PMC8507696; doi:10.3390/cancers13195029)
Supplement: Supplementary file 1 [file cancers-13-05029-s001.zip › cancers-1307301-supplementary.pdf]

Supplementary Materials

Effect of Peptide Receptor Radionuclide Therapy in Combination with Temozolomide against Tumor Angiogenesis in a Glioblastoma Model

Sang Hee Lee [1,2,†], Ji Young Choi [1,†], Jae Ho Jung [1], In Ho Song [1], Hyun Soo Park [1], Nunzio Denora [3], Francesco Leonetti [3], Sang Eun Kim [1,4,5,*], Byung Chul Lee [1,4,*]

[1] *Department of Nuclear Medicine, Seoul National University Bundang Hospital, Seoul National University College of Medicine, Seongnam, 13620, Republic of Korea*

[2] *Department of Transdisciplinary Studies, Graduate School of Convergence Science and Technology, Seoul National University, Seoul, 08826, Republic of Korea*

[3] *Department of Pharmacy – Pharmaceutical Sciences, University of Bari "A. Moro", Bari, 70121, Italy*

[4] *Center for Nanomolecular Imaging and Innovative Drug Development, Advanced Institutes of Convergence Technology, Suwon, 16229, Republic of Korea*

[5] *Department of Molecular Medicine and Biopharmaceutical Sciences, Graduate School of Convergence Science and Technology, Seoul National University, Seoul, 08826, Republic of Korea*

†Sang Hee Lee (S.H.L.) and Ji Young Choi (J.Y.C.) contributed equally to this study

**Corresponding authors: Sang Eun Kim (E-mail: kse@snu.ac.kr; Tel. No.: +82-31-787-7671) and Byung Chul Lee (E-mail: leebc@snu.ac.kr Tel. No.: +82-31-787-2956)*

Contents

Experimental Section 2

Figures S1-S3 3

S1. Comparison of methods for measuring of tumor volume and radionuclide uptake 3

S2. A preliminary experiment of ^{188}Re-IDA-D-[c(RGDfK)]$_2$ treatments 4

S3. Immunofluorescence images with Q-dot 605-D-[c(RGDfK)]$_2$ 5

Experimental Section

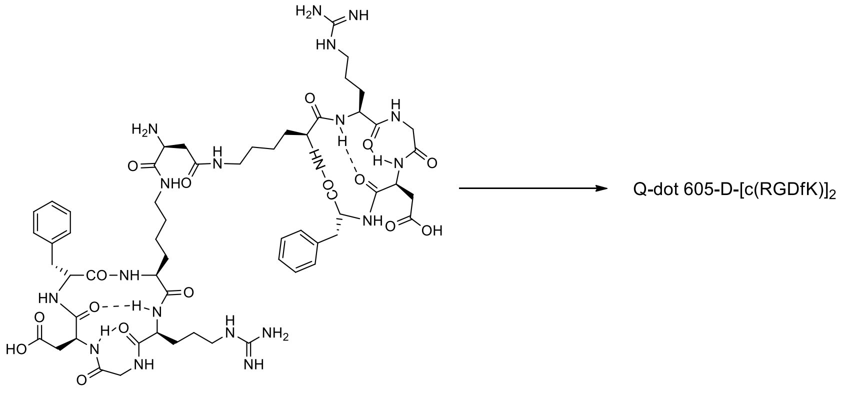

NH_2-D-[c(RGDfK)]$_2$

Synthesis of Q-dot 605-D-[c(RGDfK)]$_2$. To prepare Q-dot 605-D-[c(RGDfK)]$_2$, NH_2-D-[c(RGDfK)]$_2$ was prepared with similar method as described in our previous work [13]. NH_2-D-[c(RGDfK)]$_2$ (30 pM) was reacted with streptavidin-coated quantum dots (Q-dot 605, Qdot Corp., Hayward, CA) for 3 h at a molar ratio of 1:1 before tumor tissue labeing. After incubation, the reaction mixture was centrifuged to remove the remaining reagents. The obtained particles were washed thoroughly with 1 M tris buffer.

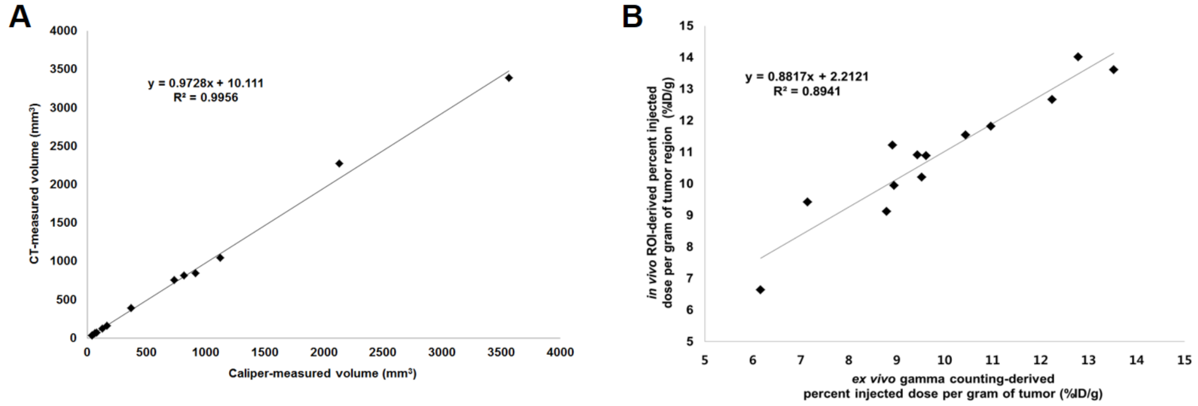

Figure S1. Comparison of methods for measuring of tumor volume and radionuclide uptake. Correlation between tumor volume measured on CT images and caliper-measured tumor volume (**A**). Animal CT measurements using an ellipsoidal the region of interest (ROI) correlated well with caliper measurements of the tumor-bearing mice: Comparisons of tumor uptake of ^{99m}Tc-IDA-D-[c(RGDfK)]$_2$ analyzed by SPECT imaging and dissected biodistribution (**B**). Measurement of ^{99m}Tc-IDA-D-[c(RGDfK)]$_2$ uptake and calculated tumor activity by *in vivo* animal SPECT analysis was compared to the true values, as measured *ex vivo* tumor tissue uptake by gamma conter.

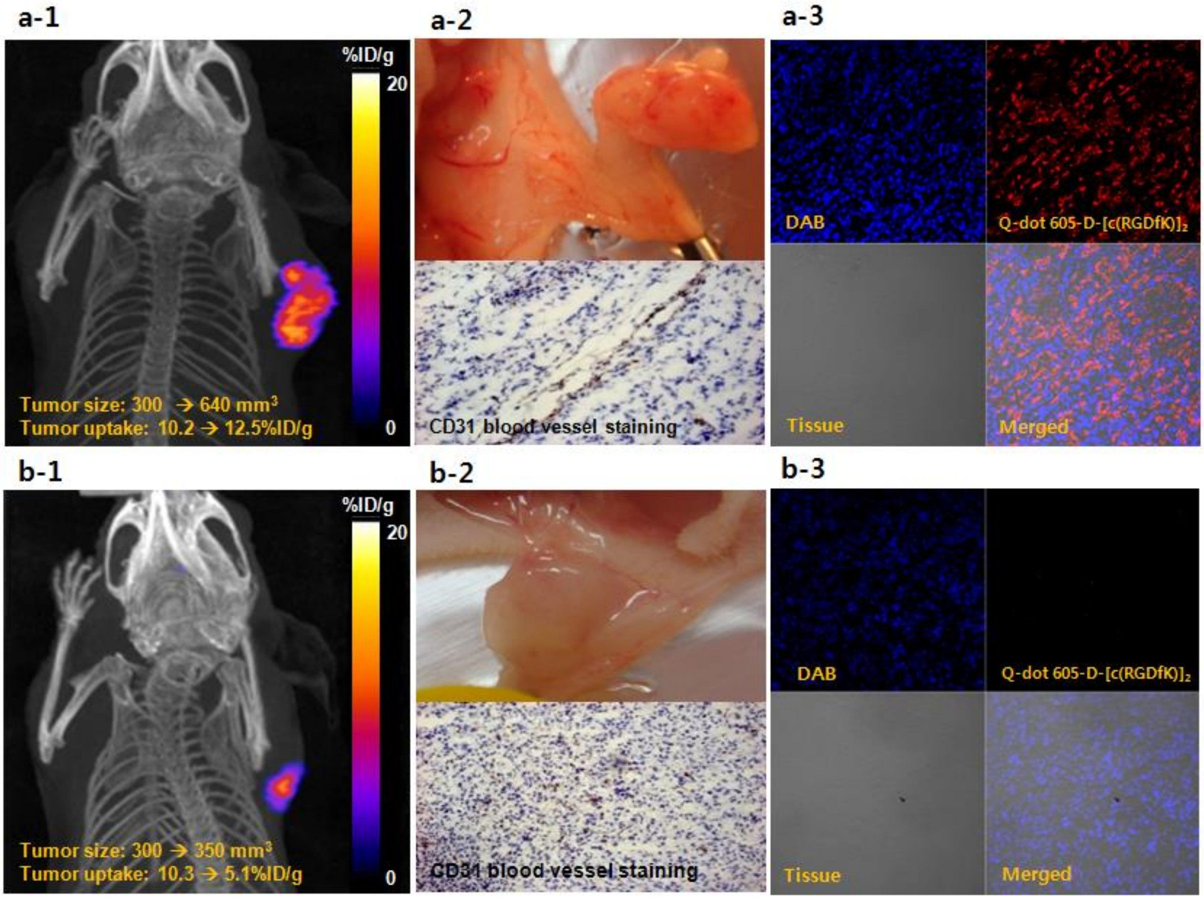

Figure S2. A preliminary experiment of ^{188}Re-IDA-D-[c(RGDfK)]$_2$ treatments in U87-MG xenografts. After tumor volume reached approximately 300 mm^3, ^{188}Re-IDA-D-[c(RGDfK)]$_2$ or saline was administered intravenously, and at 3 days postinjection thereafter, mice were sacrificed to conduct tissue distribution studies. Images are coronal view from ^{99m}Tc-IDA-D-[c(RGDfK)]$_2$ SPECT at a mid-scan time of 25 min for scan duration of 10 min after 3 days postinjection with saline and 22.2 MBq of ^{188}Re-IDA-D-[c(RGDfK)]$_2$, respectively, and showed converted values of tumor size and % ID/g in each animal (**a-1** and **b-1**). Macroscopic view of excized tumor tissues from each animal and immnunohistochemistry for microvessels in each tumor slices using an anti CD31 monoclonal antibody (**a-2** and **b-2**). Nuclei stanined with the DAB (blue), fluorescence micrographys of Q-dot-605 D-[c(RGDfK)]$_2$ (red) stained tumor slices in each animal and microscopy image of each tumor slices (**a-3** and **b-3**).

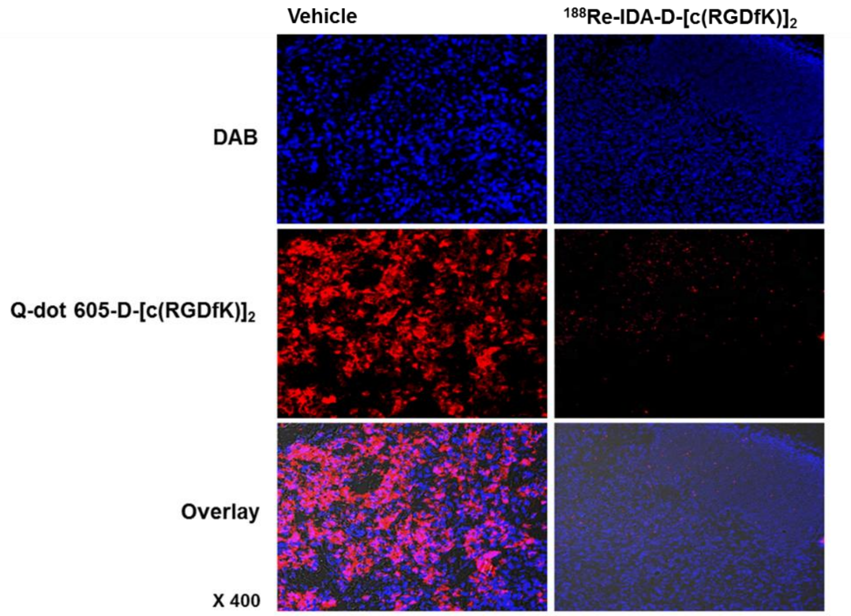

Figure S3. Immunofluorescence images with Q-dot 605-D-[c(RGDfK)]$_2$
